# Seroepidemiology of Crimean-Congo Haemorrhagic Fever among cattle in Cameroon: Implications from a One Health perspective

**DOI:** 10.1371/journal.pntd.0010217

**Published:** 2022-03-21

**Authors:** Lina González Gordon, Paul R. Bessell, Egbe F. Nkongho, Victor N. Ngwa, Vincent N. Tanya, Melissa Sander, Lucy Ndip, Kenton L. Morgan, Ian G. Handel, Stella Mazeri, Barend MdeC Bronsvoort, Robert F. Kelly

**Affiliations:** 1 Royal (Dick) School of Veterinary Studies and the Roslin Institute, University of Edinburgh, Easter Bush, United Kingdom; 2 School of Life Sciences, University of Lincoln, Lincoln, United Kingdom; 3 School of Veterinary Sciences, University of Ngaoundere, Ngaoundere, Cameroon; 4 Cameroon Academy of Sciences, Yaoundé, Cameroon; 5 Tuberculosis Reference Laboratory Bamenda, Bamenda, Cameroon; 6 Laboratory of Emerging Infectious Diseases, University of Buea, Buea, Cameroon; 7 Institute of Ageing and Chronic Disease and School of Veterinary Science, University of Liverpool, Neston, United Kingdom; Université de Montréal, CANADA

## Abstract

**Background:**

Crimean-Congo Haemorrhagic Fever (CCHF) is a tick-borne viral zoonotic disease distributed across several continents and recognized as an ongoing health threat. In humans, the infection can progress to a severe disease with high fatality, raising public health concerns due to the limited prophylactic and therapeutic options available. Animal species, clinically unaffected by the virus, serve as viral reservoirs and amplifier hosts, and can be a valuable tool for surveillance. Little is known about the occurrence and prevalence of Crimean-Congo Haemorrhagic Fever Virus (CCHFV) in Cameroon. Knowledge on CCHFV exposure and the factors associated with its presence in sentinel species are a valuable resource to better understand transmission dynamics and assess local risks for zoonotic disease emergence.

**Methods and findings:**

We conducted a CCHFV serological survey and risk factor analysis for animal level seropositivity in pastoral and dairy cattle in the North West Region (NWR) and the Vina Division (VD) of the Adamawa Region in Cameroon. Seroprevalence estimates were adjusted for sampling design-effects and test performance. In addition, explanatory multivariable logistic regression mixed-effects models were fit to estimate the effect of animal characteristics, husbandry practices, risk contacts and ecological features on the serological status of pastoral cattle. The overall seroprevalence was 56.0% (95% CI 53.5–58.6) and 6.7% (95% CI 2.6–16.1) among pastoral and dairy cattle, respectively. Animals going on transhumance had twice the odds of being seropositive (OR 2.0, 95% CI 1.1–3.8), indicating that animal movements could be implicated in disease expansion. From an ecological perspective, absolute humidity (OR 0.6, 95% CI 0.4–0.9) and shrub density (OR 2.1, 95% CI 1.4–3.2) were associated with seropositivity, which suggests an underlying viral dynamic connecting vertebrate host and ticks in a complex transmission network.

**Conclusions:**

This study demonstrated high seroprevalence levels of CCHFV antibodies in cattle in Cameroon indicating a potential risk to human populations. However, current understanding of the underlying dynamics of CCHFV locally and the real risk for human populations is incomplete. Further studies designed using a One Health approach are required to improve local knowledge of the disease, host interactions and environmental risk factors. This information is crucial to better project the risks for human populations located in CCHFV-suitable ecological niches.

## Author summary

Crimean-Congo haemorrhagic fever poses an important threat for public health, particularly in endemic locations in Eurasia and parts of Africa. Despite its long-standing recognition, CCHF is considered a neglected disease, with sporadic official case reports and evidence of serological circulation in remote rural areas and less frequently, in peri-urban settings in sub-Saharan Africa. However, the drivers associated with its emergence and the spatial-temporal patterns linked to its circulation and spread are not well-understood. In this study, we identified a high proportion of cattle with CCHFV antibodies suggestive of high levels of viral circulation in the North West and the Adamawa Regions in Cameroon. In pastoral cattle, there was a positive relationship between going on transhumance, older age, higher shrub density and lower absolute humidity and CCHFV individual seropositivity. In contrast, non-indigenous breeds reported a lower risk of being seropositive. These results can be interpreted in connection to the underlying dynamics of CCHFV cycle and underline potential mechanisms linked to disease expansion. From a public health perspective, high levels of exposure to CCHFV in cattle highlight the possibility of unrecognized human infection and therefore, emphasize on the need to remain vigilant to possible disease risks for local groups involved in pastoralism and to dairy smallholders across the region.

## Introduction

Crimean-Congo haemorrhagic fever is the most widespread tick-borne viral zoonosis of medical significance, whose pathogen, an *Orthonairovirus*, belongs to the Nairoviridae family [1,2]. Turkey, Iran, Pakistan, Russia, and Iraq have the highest burden of the disease with reports of sporadic human cases and outbreaks of different magnitude [2–4]. The rapid and growing incidence of the disease, as well as its recognized potential for emergence or re-emergence in previously naïve areas is a matter of public health concern given its infectious potential, the severity of the condition and the limited prophylactic and therapeutic options available [5,6]. As a result, in 2018, the WHO R&D Blueprint for action to prevent epidemics included CCHFV as one of the top infectious agents targeted for research and development [7].

Humans act as an incidental host and are the only known species to be clinically affected by CCHFV [8,9]. Most (~ 90%) human infections are asymptomatic or cause a non-specific mild fever with no further clinical impact [2]. Less frequently, patients develop a severe and often fatal haemorrhagic disease after a brief incubation period (~ 1 week) characterized by high-fever, fatigue, myalgia, vomiting and diarrhoea that progress to a haemorrhagic period with reports of petechiae, hematomas, generalized bleeding and multi-organ insufficiency [1,9]. The overall case fatality rate (CFR) ranges from 4.7% to 11.7%, an estimate reflecting variation related to the infectious status (ongoing, recent or past infection) [10]. Primary infection occurs after an infected tick bite, by accidental crushing of infected ticks or through direct contact with tissues, fluids or blood of viraemic hosts (domestic or wildlife animals and humans)[1,2]. Outbreaks have been generally associated with nosocomial transmission from undiagnosed cases in settings where health assistance lacks the adequate conditions to identify and isolate patients [1,11]. Otherwise, CCHF remains an occupational disease predominantly restricted to rural areas [3]; primary cases often take place among animal handlers, veterinarians and abattoir and agricultural workers, hence, these groups are identified as high-risk populations for CCHFV infection [1–3].

The disease exists in a silent enzootic cycle, whose balance relies in a complex ‘animal-tick-environment’ interplay, ultimately involving humans as accidental hosts [8]. CCHFV has been detected in a wide range of Ixodidae (hard ticks) and some Argasidae ticks (soft ticks) [10,12,13]. Gargili *et al*. (2017), reviewed the species of ticks collected from different hosts in which CCHFV has been detected [13]; *Rhipicephalus* spp., *Ixodes* spp., *Dermacentor* spp., *Amblyomma* spp., *Hyalomma* spp. are among the species implicated. However, Ixodid ticks and especially the *Hyalomma* genus have a major role as the most important reservoir and vector of CCHFV and its presence closely approximates high-risk areas [13]. Landscape fragmentation, climate variations and shifts in the distribution or abundance of the main vectors and amplifying hosts affect the viral cycle; therefore, it has been proposed that CCHFV dynamics are strongly influenced by ecological variation [14,15]. Ultimately, these interactions have implications on viral seasonal patterns reported in endemic locations and can modify the likelihood of disease emergence and re-emergence in other settings [3,14–17].

Under this rationale, sero-surveys in domestic and wildlife species have been proposed as a powerful tool to estimate the potential risk that CCHFV poses to a region [18,19]. Livestock, in which CCHFV causes minor or no clinical disease, coexist closely with human populations and serve as a valuable species for surveillance purposes [12,19,20]. In addition, ecological analysis based on the presumed distribution of CCHFV in animals can provide insights on hidden disease dynamics, a resource worthwhile exploring in areas with scarce information on the disease distribution in human populations alongside reports of suitable vectors and ecological drivers that would facilitate disease emergence.

The *Hyalomma* genus of ticks is widely distributed across Africa [21]. However, despite the general lack of CCHF clinical case reports from Central Africa, with the exception of the Democratic Republic of the Congo [22], large geographical areas across the continent offer suitable tick habitats and the potential for CCHFV circulation and human outbreaks [4,23]. Most countries in Africa which have documented CCHFV serologically in livestock (including Sudan, Mauritania, South Africa, Egypt, Zimbabwe, Senegal and Uganda), have also documented CCHF cases in humans [4,24–35]. In contrast, other countries (including Niger and Guinea) have reported evidence of CCVFV circulation in animals, humans or tick vectors without official reports of clinical disease to date [36,37].

Cameroon, located in Central-Western sub-Saharan Africa, fulfils the requirements for a silent CCHFV cycle as already described in other settings: vector presence, availability and distribution of amplifying hosts, evidence of viral circulation and is adjacent to countries already identified to have endemic infection cycles [4,38,39]. In fact, recent estimations from global CCHFV ecological niche models suggest the possibility of CCHFV emergence in Cameroon based on viral and tick ecological suitability [23,39]. However, field-based epidemiological studies designed to assess local distribution in at-risk populations and reservoirs are scarce. On-site studies have the potential to improve the understanding of viral patterns providing baseline information that can be later used for public health advocacy and to guide further epidemiological analysis aimed to accurately map the risk of CCHFV transmission in human populations.

In this study, we look to maximise the value of available epidemiological information and biobanked cattle serum samples (with associated metadata) to gain disease understanding preceding the report of human cases. We estimate CCHFV seroprevalence and present the results of an epidemiological risk factor analysis of CCHFV seropositivity in cattle in two of the major cattle keeping areas of Cameroon, the North West Region and the Vina Division of the Adamawa Region. Finally, we discuss the potential public health implications of our findings in Cameroon from a One Health perspective.

## Materials and methods

### Ethical statement

The Institute of Research and Development (Cameroon) and The University of Edinburgh Ethics Committee (UK) approved the study at the moment of data collection (VERC No: OS02-13). Verbal permission was obtained from all herdsmen in order to collect the biological samples from the animals and before administering the questionnaire. A brief explanation of the purpose and procedures of the study preceded the consent and herdsmen were informed of the possibility of opting out at any stage. The Roslin Institute, at the Royal (Dick) School of Veterinary Sciences, University of Edinburgh, UK, approved the serological assessment of CCHFV antibodies in the serum bank in 2019.

### Background and study setting

**Fig 1:**
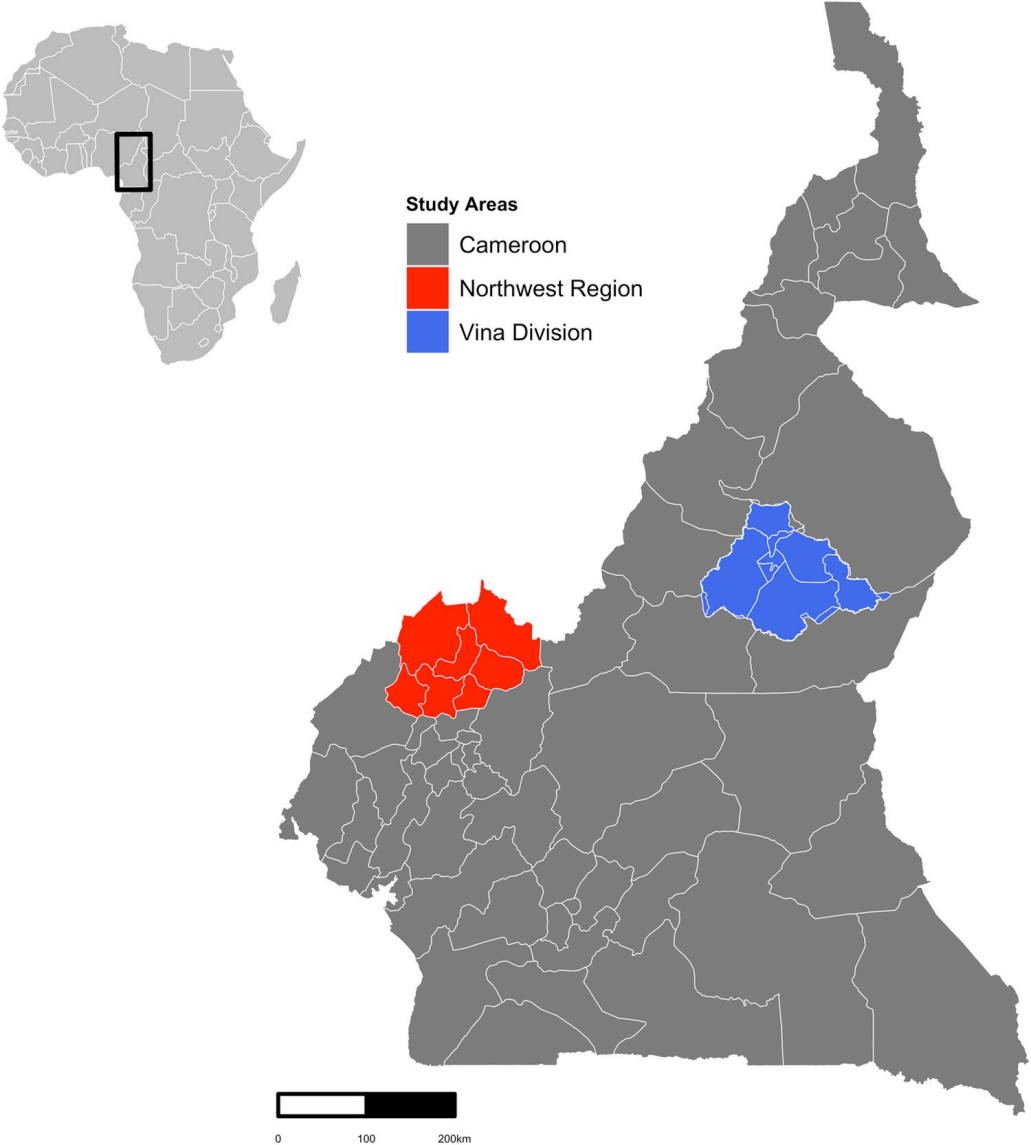
Map of Cameroon depicting the study regions in the North West Region and the Adamawa Region. The red area shows the North West Region and its Divisions. The blue area shows the Vina Division of the Adamawa Region and its sub-Divisions. Shapefile obtained from GADM database, freely available for academic uses with permission from Global Administrative Areas (https://gadm.org/maps/CMR.html). The figure was made with RStudio version 3.5.3.

Cameroon is an ecologically diverse country in Central Africa covering an area of 475,440 km^2^. It borders Nigeria, the Republic of the Congo, Gabon, Equatorial Guinea, Central African Republic, and Chad as well as a coast on the Gulf of Guinea. Climate dynamics can be generally thought of in terms of a wet (May–October) and a dry period (November–April) with rainfall and temperature varying monthly according to the season [40,41]. The country is organized in 10 administrative Regions; these Regions are further split into Divisions and sub-Divisions [42]. Major cattle producing areas are the North West, North, Extreme North, northern parts of the East and Central, and the Adamawa Regions. Overall, the cattle population has increased over recent years and the country is a recognized livestock producer in the Central-West African region [40,43]. The current analysis is focused on data collected from animals reared in the North West Region (NWR) and Vina Division (VD) of the Adamawa Region (Fig 1).

The Adamawa Region occupies a 64,000 km^2^ territory, generally located over 1,200 m and classified as Guinea savannah, characterized by woodland and grass savannah vegetation [41,44]. Rearing cattle is the main economic activity of the region and it is focused on pastoralist systems primarily managed by local ethnic groups; however, some residents are crop growers working under the principle of a collaborative agricultural economy [44]. Within the Adamawa Region, the VD has a land area of 17,196 km^2^ with altitudes ranging between 500–2,500 m; the area is topographically characterized by a mountainous western border that softens as it reaches east into an undulating grassland savannah [40]. Similarly, the NWR occupies an area of 17,300 km^2^ with distinctive rocky-mountains rising between 700 and 3,000 m, although subtropical forest and plateaux savannah are also present [40]. In terms of economy, the agricultural sector is strong and represents the main source of income in the rural areas (~ 80%). Furthermore, it is estimated that 60% of the NWR is a viable terrain for livestock production leading to an active involvement in beef farming [45].

Fulbé, Mbororo, Niam Niam, Laka, Mboum and Baya ethnic groups populate the North West and Adamawa Regions. Some of them (e.g. Fulbé and Mbororo), are part of the Fulani ethnic group, widely extended across sub-Saharan West and Central Africa and recognized as the main pastoral community [40]. In 2013, the cattle populations of the NWR and VD were estimated to be 546,508 and 176,257 respectively, with herd sizes ranging from 50 to 150 cattle [40]. The predominant breed is the *Bos indicus* Fulani cattle but other improved breeds such as the Gudali and crossbreeds are also widespread particularly in the Adamawa Region. The latter breeds provide either better productivity or resilience against the harsh conditions of the territory [40]. Cattle are normally grazed in an extensive system on communal pastures close to the farm [40,45]. During the dry season, transhumance, a pastoral practice involving long-distance movement of animals takes place, with the aim of overcoming the seasonal shortages in pasture availability [46]. Intensive farming systems for cattle production are not a common practice. However, semi-intensive farming, primarily based on a cut-and-carry feeding system, is of growing importance for the dairy sector where imported Holstein-Friesian crosses are used. But this remains a very small proportion of the livestock industry in the country [45].

### Study design

Pastoral herds located across the NWR and VD and dairy herds from the NWR were studied to estimate CCHFV antibody prevalence based on serum bank samples available from two previous cross-sectional studies investigating the epidemiology and phylogenetics of bovine tuberculosis and liver fluke infections in Cameroon [40]. Pastoral cattle located at the NWR and VD comprised the main sampling frame, which was built based on the official vaccination records for 2012 (Sampling frame: 5,053 pastoralists’ herds). Conversely, dairy herds from the NWR were retrieved and sampled based on data from the three largest dairy cooperatives registered for the area in 2012 (Sampling frame: 164 dairy herds). A herd was defined as an established group of animals managed collectively as a unit under a well-structured ownership model. In both cases, the Ministry of Livestock, Fisheries and Animal Industries (MINEPIA) provided the records as the closest representation of the true number of herds according to an official source. Sampling and data collection took place from January to May 2013 in the NWR and from September to November 2013 in the VD.

The list of pastoralist herds in each site was stratified by administrative area; seven Divisions in the NWR and eight sub-Divisions within the VD. A random sample of 50 herds was taken proportional to the total number of herds listed per study site in the NWR and the VD. In each herd, 14–15 animals were selected by quasi-random sampling, stratified to three age classes: 6 months– 2 years-old, 2–5 years-old and >5 years-old, termed young, adult, and old groups, respectively. Likewise, a stratified random sample of 46 small-scale dairy herds was selected proportional to the number of dairy herds per cooperative in the NWR; all animals were sampled per herd (one to three animals per smallholder). Herd replacement by resampling was applied in both scenarios if herdsmen were unwilling to engage with the study or when unforeseen logistical situations prevented visiting one of the originally selected herds. In-depth information about study design, sample size calculations and sampling methods used in the original study has been reported by Kelly *et al*. (2016) [47].

### Serum biobank and associated cattle metadata

Biobanked cattle serum samples were available for processing at the Roslin Institute at the Royal (Dick) School of Veterinary Medicine (University of Edinburgh), UK. Before transportation to UK, all samples underwent water-bath heat treatment for 120 minutes at 56°C. Serum samples were labelled to allow linking back to herd and animal level questionnaire data and stored at -20°C until processed. Individual and herd level data was available from a structured questionnaire administered to each herdsman in Fulfulde. The questionnaire covered aspects of cattle husbandry and management, dairy practices, individual animal features and GPS location of the farm. A copy of the questionnaire and further details on data collection are reported by Kelly *et al*. (2016, 2021) [47,48]. Age was estimated by dentition score according to the number of permanent incisor teeth present and classified from < 2 years (no permanent incisors) to ≥ 5 years (all incisors in wear/broken). Calves defined as less than 6 months old by the herdsman were excluded from the sampling to avoid potential issues of maternal antibodies. Breed was defined according to phenotypic traits as either mixed breed (mixed *Bos indicus*), exotic (any pure *Bos taurus* or crossbreeds *Bos indicus* x *Bos taurus*), Fulani and Gudali [40]. While Fulani and Gudali are considered local breeds, the Gudali cattle has been systematically improved through a government-led programme over the last 50 years [49]; therefore, Fulani was considered the indigenous breed for the study area.

### Laboratory testing

Laboratory analysis was carried out in February and March 2019 at the facilities of the Roslin Institute at the Royal (Dick) School of Veterinary Medicine (University of Edinburgh), UK. Anti- CCHFV Immunoglobulin G screening (IgG) was performed by a commercial double-antigen Enzyme Linked Immunoabsorbant Assay (ELISA) (ID.vet, Grabels, France) based on the recombinant nucleoprotein antigen (NP), with a reported diagnostic sensitivity of 98.9% (95% CI 96.8–99.8) and specificity of 100% (95% CI 99.8–100) [50]. The test was performed in 96-well microplates following the manufacturer´s instructions [51]. Duplicate kit positive and negative controls were included per plate. Optical density (OD) values were determined spectrophotometrically using an automated ELISA reader (Themocientific Multiskan Go) set at 450 nm. The validity of the test results was verified for every plate based on the manufacturers recommendations of an OD for the positive controls > 0.35 and a ratio of the mean OD values of the negative and positive controls > 3. The calculation of the S/P% as the ratio of the sample OD and the mean OD of the positive controls of the plate was used as the output measure and an S/P percentage >30% were recorded as positive. Quality control was performed for a total of 180 samples (2 plates), with 100% concordant results. Individual S/P% values were collected per processed plate by using a standard report datasheet and combined into a final database.

### Data analysis and statistics

All data analyses were performed using R packages and functions in RStudio version 3.5.3 [52,53]. Figures, graphs and maps were plotted using the *ggplot2* package [54]. The shapefiles of the country maps and its administrative divisions were obtained from the open access database of Global Administrative Areas (GADM) [55].

#### Prevalence estimation

A design effect correction, accounting for the stratified population structure of the pastoral cross-sectional study was implemented [56]. Clustering (herd identification), strata information (Divisions and sub-Divisions) and animal and herd sampling weights were combined into a nested complex survey object using the *svydesign* function in the *Survey* package [56]. This survey object was then used within the package’s summary functions to obtain CCHFV survey design-adjusted seroprevalence estimates. Thereafter, seroprevalence values in pastoralist and dairy herds were corrected for test performance using the Wilson’s method to provide appropriate confidence intervals for the adjusted seroprevalence, while accounting for the imperfect test sensitivity and specificity [57].

#### Individual risk model and ecological analysis

Within the pastoralist subset, two separate explanatory multivariable mixed-effect logistic regression models were used to estimate the effect of individual animal features, risk contacts, animal husbandry practices and environmental variables on CCHFV serological status of cattle. Both herd and administrative Division or sub-Division were included as random terms to account for the clustering effect of the sampling design. Global models were built using all the selected fixed effects through the *glmer* function in the *lme4* package [58].

The first model explored the effect of individual traits, risk contacts and animal husbandry practices (“Individual risk-factor model”). Preliminary variable selection considered central biological or epidemiological attributes related to disease risk as per its potential causal dependency network. Statistically significant variables based on univariable analysis with a cut-off value of p≤0.2 were also included [59]. A multi-correlation matrix was used to check for the presence of highly correlated explanatory variables. Multi-model inference was performed in order to reduce variable selection bias, achieve a better precision and approach model selection uncertainty. Model averaging was used to estimate the final coefficients using the *MuMIN* package [60]. A subset of models was generated based on all the potential combinations of the fixed effects considered at the global model; each candidate model was assessed by the delta Akaike´s Information Criterion (AIC). Candidate models with a delta AIC (Δ_i_) ≤ 2 were averaged, as they are considered to be as good as the best model [61].

The second model focused on the role of ecological covariates (“Ecological model”) on CCHFV seropositivity by means of a standard logistic regression model adjusting for the influence of significant features identified through the Individual risk-factor model. Animals that went on transhumance were purposively removed from the analysis to reduce the possibility of bias introduced by animal movement on the inference of ecological features for past disease exposure.

Elevation at the georeferenced point was extracted from an SRTM30 digital elevation model (DEM). Modelled climate data was downloaded from the UEA Climate Research Unit (version 4.03)[62]. The mean of the mean, minimum, maximum temperature, vapour pressure (used to calculate absolute and relative humidity), and precipitation for the years 2011–2014 were extracted at the location of each georeferenced point. To test the immediate weather impacts on seropositivity, we compared the climate average of these to the mean values during a 90-day window prior to sampling. Landcover variables were downloaded from the European Space Agency (ESA) climate change initiative (CCI) landcover classification [63]. To describe the landcover in the area surrounding the farm, the number of pixels of grassland, shrubland and trees within 5 km of each point were extracted. Climate and spatial variables were rescaled to aid model fitting as required. Model selection was performed through the AIC criterion.

Final model estimates were converted from the log scale to OR and presented in conjunction with its 95% CI. Model diagnosis was conducted through visual inspection of plots and goodness-of-fit measures for hierarchical regression models with a binary outcome; residual diagnosis, coefficient of determination (D) and ROC curves were assessed [64–66]. In addition, the Intraclass Correlation Coefficient (ICC) was calculated to indicate the proportion of variance explained by the clustered study population [67]. Details on the procedures used for model selection and assessment of model performance and fit are documented in S1 Appendix (“Individual risk-factor model”) and S2 Appendix (“Ecological model”).

## Results

### Study population characteristics

A detailed description of cattle features and herd husbandry practices has been previously reported by Kelly *et al*. (2016)[47]. A brief overview of the population characteristics is included here as relevant background information and to support the interpretation of the results. In total, 1,498 cattle were analysed as part of the pastoral population with 71.2% (95% CI 68.9–73.5) of the sample comprised by females. There was a difference in the breeds identified per study location; in the NWR, 64.6% (95% CI 61.2–68.2) of cattle were mixed breed, whereas Gudali was the predominant breed reared in VD (82.4%, 95% CI 79.9–85.2). No difference in age distribution was identified across study locations (χ^2^-test, p > 0.05). The majority of herds reared animals in an open grazing system with access to natural pasture (90.9%, 95% CI 89.5–92.4); thus, contact with domestic and wildlife species, as well as with other herds was commonly documented. Almost a quarter of the cattle population went on transhumance (24.9%, 95% CI 22.7–27.1). However, 44.0% (95% CI 40.4–47.5) of animals from the NWR were involved with the practice, much more often than the animals reared in the VD (5.8%, 95% CI 4.1–7.5). In comparison, the 60 animals sampled within the dairy herds represented a mature (Adult: 49.1%, 95% CI 35.1–63.2 and Old: 43.4%, 95% CI 29.8–57.7), non-indigenous Holstein-Friesian (98.3%, 95% CI 91.1–99.9) and non-transhumant population. Around 95% (95% CI 86.1–98.9) of the animals were kept housed most of the time and 8.3% (95% CI 2.8–18.4) were allowed to graze in open pastures. Contact with domestic animals, especially birds, dogs, sheep and goats was frequent; in contrast, contact with wildlife species was not reported.

### Pastoral cattle

#### CCHFV prevalence and spatial distribution of seropositive animals

**Fig 2:**
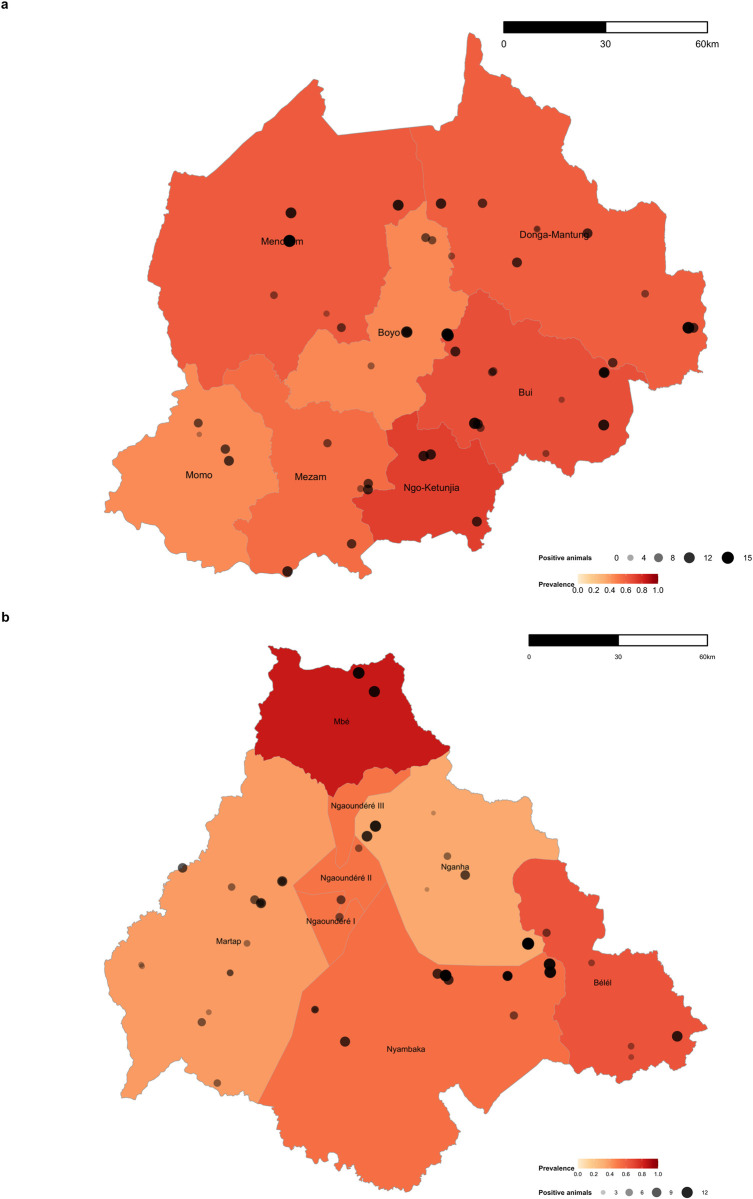
Crimean-Congo Haemorrhagic Fever Virus seroprevalence at the Division level within the North West Region and at the sub-Division level within the Vina Division of the Adamawa Region. The increasing gradient in the coloured areas indicates a higher seroprevalence according to the total of animals analysed for the location. Sized bubbles indicate the number of seropositive animals within each sampled herd. Total sample size: 750 animals (50 herds) at the NWR (a) and 748 animals (50 herds) at the VD (b). Shapefile obtained from GADM database, freely available for academic uses with permission from Global Administrative Areas (https://gadm.org/maps/CMR.html). The figure was made with R studio version 3.5.3.

Serological analysis revealed that 807 out of the 1,498 pastoral animals had been exposed to CCHFV, with an adjusted seroprevalence of 56.0% (95% CI 53.5–58.6) for the entire population. A slightly higher seroprevalence was estimated in the North West Region (57.9%, 95% CI 54.4–61.5) in comparison to the Vina Division (48.9%, 95% CI 45.3–52.6). The Divisional and sub-Divisional seroprevalence values were heterogeneous (Fig 2; seroprevalence calculations per location shown in S1 Table).

Analysis of the seroprevalence by herd identified a widespread distribution of the cases with 98.0% (95% CI 92.9–99.7) of the herds with at least one seropositive animal. Within the NWR, 22/50 herds (44%) reported 10 or more seropositive animals, whereas, for the VD this proportion was 17/50 sampled herds (34%).

#### Risk factors for animal-level seropositivity to CCHFV

Final results for the averaged “Individual risk-factor model” for the pastoral sub-population revealed that age, breed and going on transhumance were associated with past exposure to CCHFV (Table 1). Contact with other species including dogs, sheep, goats, hogs, buffaloes and antelopes did not influence the probability of individual exposure in our sample as indicated through herdsmen reports of cattle contacts. Adult animals had a higher risk of exposure when compared to young stock, with the oldest animals showing the highest likelihood of being seropositive (OR: 29.0, 95% CI 19.3–43.7). In addition, non-indigenous breeds (Gudali or crossbreeds) were less likely to have circulating antibodies against CCHFV compared to Fulani cattle (OR 0.6, 95% CI 0.4–0.9). Lastly, we identified that animals with a history of going on transhumance had twice the odds of being seropositive (OR: 2.0, 95% CI: 1.1–3.8) when compared to those not involved in the practice. Cattle purchase report was not identified as a risk factor for seropositivity. The AUC for the averaged model was 0.872, which indicates that the model discriminates well between seropositive and seronegative animals. Furthermore, around 28% of the variation in the results is explained by the hierarchical structure of the studied population, as shown by the adjusted ICC (ICC_*adj*_) across the candidate models (See S1 Appendix). The latter measure explores the importance of the contextual background of cattle when modelling CCHFV seropositivity. ICC_*adj*_ accounts for the relatedness (non-independence) of animals within herds located in the same area as similarities in geographical conditions, husbandry practices and vector distribution have the potential to alter the probability of infection within a cluster. Additional information on model performance is available in S1 Appendix.

**Table 1:** Multivariable averaged-mixed effects logistic regression model for Crimean-Congo Haemorrhagic Fever Virus seropositivity in pastoral cattle.

Variable	OR (95% CI)	Sum of weights^†^
**Sex**		0.08
Female	Reference 0.99 (0.89–1.10)	
Male		
**Breed**		1
Fulani	Reference 0.59 (0.37–0.95)	
Gudali /Crossbreed		
**Age**		1
Young	Reference 3.29 (2.42–4.48) 29.06 (19.31–43.73)	
Adult		
Old		
**Buying cattle**		0.82
No	Reference 1.47 (0.80–2.67)	
Yes		
**Going on transhumance**		1
No	Reference 2.01 (1.07–3.81)	
Yes		
**Contact with dogs**		0.08
No	Reference 1.01 (0.84–1.21)	
Yes		
**Contact with sheep**		0.21
No	Reference 1.07 (0.73–1.55)	
Yes		
**Contact with goats**		0.06
No	Reference 0.99 (0.86–1.15)	
Yes		
**Contact with buffaloes**		0.09
No	Reference 1.03 (0.68–1.56)	
Yes		
**Contact with hogs**		0.07
No	Reference 0.98 (0.82–1.18)	
Yes		
**Contact with antelopes**		0.07
No	Reference 1.01 (0.85–1.19)	
Yes		
**Study location**		0.07
North West Region	Reference 1.01 (0.81–1.24)	
Vina Division		

^†^ Measure of the relative importance of each variable in the final averaged model, calculated as the sum of model weights over all models (n = 11) containing each explanatory variable.

Absolute humidity and shrub density were the only two examined factors strongly associated with CCHFV antibody seropositivity in pastoral cattle that did not go on transhumance. These results were obtained after adjusting for the effect of age and breed, features already linked to seroprevalence through the individual risk-factor model (Table 2). The proportion of shrubland within a 5 km radius increased the odds of seropositivity (OR 2.1, 95% CI 1.4–3.2), whereas animals located in areas of higher absolute humidity had decreased odds of CCHFV exposure (OR 0.6, 95% CI 0.4–0.9). The grouping structure of the population explained approximately 20% of the variation in the exposure to CCHFV, as indicated by the ICC_*adj*_. The model performed well; its discriminatory power (as quantified by the AUC) was 0.862. Additional information on model performance is available in S2 Appendix.

**Table 2:** Multivariable logistic regression model for Crimean-Congo Haemorrhagic Fever Virus seropositivity in non-transhumant pastoral cattle.

Variable	OR (95% CI)
**Age**	
Young	Reference 3.13 (2.19–4.45) 25.3 (16.1–39.6)
Adult	
Old	
**Breed**	
Fulani	Reference 0.48 (0.28–0.83)
Gudali /Crossbreed	
**Study location**	
North West Region	Reference 1.13 (0.49–2.66)
Vina Division	
**Shrubland**	2.12 (1.42–3.18)
**Absolute humidity**	0.58 (0.38–0.90)

### Dairy cattle

#### CCHF prevalence and spatial distribution of seropositive animals

The seroprevalence of antibodies against CCHFV in dairy cattle was 6.7% (95% CI 2.6–16.1), equivalent to 4 animals, each from a different herd (herd location and serological status shown in S1 Fig). No statistically significant difference was found between animal features (demography, husbandry, and risk contacts) and CCHFV serological status of dairy cattle (Fisheŕs test, p>0.05). Multivariable modelling was not conducted due to the low number of positive animals.

## Discussion

Livestock species have a recognized role in CCHFV epidemiology; besides harbouring ticks with the potential to maintain and transmit CCFHV, they also serve as efficient amplifier hosts [19,68]. As a result, livestock are often included as part of surveillance initiatives and outbreak investigations in endemic and non-endemic areas [18,19]. In this survey, we found a high seroprevalence (~56.0%) of antibodies in pastoral cattle, which contrasts to the lower seroprevalence detected among the dairy population (~6.7%). A prior report documented the circulation of CCHFV in the country (2013–2014) using cattle samples collected in the Extreme North, North, Adamawa, Central and South Regions; this study showed an overall seroprevalence of 74% [69], a much higher estimate in comparison to the overall findings of our survey. The variation in IgG levels may be due to differences in the population, study design and analysis including discrepancies in the age structure and tick infestation of animals, sampling methods and locations, diagnostic test performance or data processing.

In Cameroon, pastoral and dairy cattle are managed very differently, which is likely to greatly alter their baseline risk of exposure to tick-borne pathogens such as CCHFV. Not many studies have focused on analysing viral circulation in dairy herds; however, our results were comparable to previous analyses that observed low CCHFV seroprevalence in dairy animals. For instance, in high-disease burden scenarios in Iran, serological assessments among dairy cattle have revealed less than 10% CCFHV reactivity [70,71]. In the NWR, smallholder dairy keeping is characterized by regular and closer contact with cattle. Dairy cattle were largely housed compared to the extensive rearing system in the traditional pastoralist herds. As a result, this semi-intensive production system allows fewer opportunities to graze and little contact with other domestic and wildlife species, which could act as a protective factor for tick infestation. Despite a presumed low risk of tick infestation under these conditions, ticks can still be a problem in ‘zero-grazed’ systems [72]. For example, ticks can be carried in on cut grass or may be introduced by new stock. Moreover, in zero-grazed systems, tick infestation rates vary, among others, according to the location, season, breed, sex, age group and other features linked to individual fitness [72–74]. The interaction between a large number of factors relating to both the tick vector and host may be important for viral maintenance and, although we have shed some light on these here, further work from longitudinal studies is needed to understand how and when animals are getting infected.

CCHFV endemicity is linked to the distribution and population dynamics of ticks which have the potential to act as competent vectors. While *Hyalomma marginatum* is the main vector and reservoir for CCHFV, many other tick species are known to be involved in its epidemiology [10,13]. In Cameroon, cattle-associated tick populations include *Amblyomma* spp, *Rhipicephalus* spp. and *Hyalomma* spp. ticks which are established in different agro-ecological zones across the country [75,76]. Although *Hyalomma* spp. ticks are not the most prevalent tick genera; *Hyalomma impeltatum*, *Hyalomma impressum*, *Hyalomma rufipes* and *Hyalomma truncatum* are all encountered by cattle in Cameroon [75,76]. CCHFV RNA has been detected in *H*. *truncatum* ticks in Cameroon [69], illustrating local viral circulation at the vector-level. Therefore, it is possible that other tick species currently act as CCHFV vectors and reservoirs and are capable of maintaining viral circulation in Cameroon [10,13]. However, this study was based on biobanked sera from a tuberculosis-related study and detailed tick infestation data was not available. Information on tick abundance, species diversity and its CCHFV status would have contributed to understanding the disease ecology in the studied area. Despite this limitation, the models revealed several individual and ecological factors that are associated with CCHFV seropositivity in cattle which suggest ongoing processes at the tick-virus-host interface. These include the role of transhumance on CCFHV infection, possibly facilitated by tick exposure in the transhumance routes and, the effect of climatic and land-cover variables on CCHFV past exposure in cattle.

As it is the case for many vector-borne pathogens, host factors are important when analysing the evidence of serological exposure to CCHFV. The occurrence of antibodies against CCHFV in cattle in African countries has shown to be heterogenous and context-dependent. The effect of age, sex, tick infestation rates, animal purpose, grazing system, herd size, vector control strategies, and presence of comorbidities on CCHFV reactivity has been discussed in earlier studies [27,77,78]. Less frequently, studies have documented the association of landscape and climatic features on CCHFV antibody presence in livestock [79]. In our study, breed was connected to variations in seropositivity. Breed susceptibility to ticks, natural tick infestation rates and husbandry practices might be a contributing factor for this difference. Fulani cattle have a higher tick infestation burden, harbouring several tick species in comparison to the low tick burdens reported in Gudali cattle [80,81]. Herdsmen in Cameroon generally practice hand-removal of ticks as few areas have functioning dip tanks or access to acaricides. As a result, smaller ticks, nymphal stages, and ticks in inaccessible body locations (e.g. groins, hooves, mammary gland, tail) are usually left unattended [82]. In practice, this means that several tick species including *Rhipicephalus* spp., *Amblyomma variegatum* and *Hyalomma* spp., all potential CCHFV vectors with known presence in Cameroon [75,76], can easily remain attached long-enough to increase the probability of transmission [13].

Furthermore, animal’s age (being older) was strongly associated with an increased likelihood of the animal being seropositive in pastoral cattle. Age is a recognized risk factor for many vector-borne diseases in animals and in humans and consistent results are also available for CCHFV infection in livestock [26,27,35,83]. Higher tick infestation rates, typical of adult cattle in African settings and therefore, an increased likelihood of coming into contact with an infected tick over time might translate into an increased probability of CCHFV infection, seroconversion and thus, antibody seropositivity [82].

Importantly, our analysis highlights the potential role of the seasonal transhumant movements on CCHFV increasing infection risk. Transhumance is a socio-cultural husbandry practice of pastoralist communities where herds move to river valleys during the dry season in search of better pasture. In Cameroon, transhumant herds can travel over 600 km across regions or international borders potentially passing through areas diversely suited for several tick species and directly connecting populations that would otherwise be spatially separated [46]. CCHFV naïve cattle can get infected along the way through the uptake of ticks from remote areas, but equally likely ticks can also spread to transhumance sites, a phenomenon already documented in Africa [3,84]. Similar mechanisms have been linked to the transboundary expansion of CCHFV through small ruminants and birds [85], and are known to contribute to the transmission of other tick-borne diseases in livestock [86].

The patterns and distribution of CCHFV can also be influenced by climatic, environmental and landscape differences due to their effect on the tick vector. The ecological model suggested that CCHFV seropositivity increased among cattle using areas of increased shrub-density, whereas locations with higher absolute humidity were associated with a decreased odds of CCHFV seropositivity. Water vapor uptake is fundamental to maintain water balance in ticks and directly influences their survival rates [87], and so, water vapour deficit (WVD) is thought to impact tick developmental rates [14]. As a result, it has been used to model environmental restrictions for *H*. *marginatum* and other hard-bodied ticks [14]. The inverse relationship between absolute humidity and CCHFV seropositivity that is indicated here, challenges the results of previous reports in which ticks tend to thrive preferentially in humid areas [21]. But it should be noted that there was no association with relative humidity in our analysis. Several studies document a positive association between high humidity and CCHF incidence in humans through the retrospective analysis of cases in endemic settings [88,89]. However, it is important to emphasize that in Cameroon the understanding of CCHFV is limited, particularly in the microhabitats in which the ticks exist rather than the 0.5° resolution of the climate data. Furthermore, a great deal of uncertainty surrounds the hosts and tick vectors involved in the viral epidemiological cycle at the local level. Some explanations for the inverse relation between absolute humidity and antibody presence in cattle are possible. From a biological perspective, several tick species display a great degree of environmental and climatic adaptation which is formally recognized as ‘ecological plasticity’ [90]. Consequently, it is possible that a lower absolute humidity is not an impediment for tick establishment in the studied setting. Further knowledge on the tick species inhabiting this area is necessary to clarify how well ticks adapt to the local conditions and if they indeed act as competent vectors for CCHFV. In contrast, from a methodological perspective, the lag time between the ecological exposure (humid climate) and the serological outcome might have played a role in this relationship. The exact time of CCFHV infection cannot be inferred based on serological evidence of past exposure to CCHFV, hence, the association might not be reflecting the ecological conditions at the time of exposure to the virus. This misclassification, common when inferring cross-sectional associations, cannot be ruled out as a plausible explanation for the unexpected link between absolute humidity and serological evidence of CCHFV in pastoral cattle [91].

Furthermore, tick abundance seems to be strongly related to tree species composition and shrub cover [92,93]. Some prior geo-spatial analyses on CCHFV occurrence have indicated a connection between land fragmentation, shrubby/grassy vegetation, and tick distribution in highly affected locations [94,95]. A higher proportion of grass and shrub land cover account for almost 62% of CCHFV predicted emergence or re-emergence in humans according to an estimates obtained from a global CCHFV distribution model; land surface temperature, vegetation index and annual precipitation also contributed to the overall effect [39]. Vegetation structure might be an indicator of wildlife ecological niches supporting tick populations. Shrubby areas tend to be rich and diverse in small mammals. Therefore, the increased risk of CCHFV exposure in cattle could be better explained based on the wildlife-livestock interface enabled by open grazing systems adopted by pastoralists in the two study sites.

Contact between cattle and other domestic and wildlife species is usual in pastoralist systems. However, despite reports of different wildlife ungulates commonly grazing together with cattle in the North West and Adamawa Regions, we did not find an association of cattle contact history and CCHFV antibody presence. Yet, contact with wildlife and domestic species remains a possible source of CCHFV, especially in open-grazing, transhumant systems enabling a diverse configuration of host-vector-host interactions. Evidence collected from serological studies across Asia, Africa and Eastern Europe suggests that several domestic and wildlife species are involved in CCHFV ecology and can modulate local transmission patterns [19]. For instance, prior exposure to CCHFV has been reported in African warthogs (*Phacochoerus aethiopicus*), a species observed on the transhumance routes of the Adamawa Region [19,41]. Given the rich faunal inventories in Cameroon, the involvement of a number of endemic small mammals, birds and larger ungulates in the infection cycle, if any, is yet to be elucidated. Similarly, the participation and pathways in which other domestic animals and tick species contribute to CCHFV epidemiology merits further attention.

Our research, based on CCHFV antibody identification in cattle, highlights a potential public health risk to livestock-rearing communities and slaughterhouse workers in Cameroon. Nonetheless, further studies are required to explore the significance of CCHFV circulation in cattle for humans co-existing in this environment. To the best of our knowledge, there are no reports of CCHF clinical cases in human populations in Cameroon [4,10,31–33]. However, the infection is known to circulate at a low prevalence in rural and peri-urban areas [38,96]. Traditional communities might be at greater risk of infection. For instance, CCHFV exposure has been identified among pygmies located at the rain forest in Eastern Cameroon [38]. These communities are hunter-gatherer groups whose high-risk practices and close proximity to CCHFV-suitable ecological niches probably contributes to viral transmission. In parallel, pastoralist communities at the North West and the Adamawa Regions might engage in high-risk practices leading to contact with tissues, fluids, and blood from viraemic livestock or virus-carrying ticks. Manual-tick removal is one example of possible infection routes. Similar risks are transferable to dairy farmers; in the past, CCHFV circulation in dairy cattle has been linked to human outbreaks [97]. Thus, the potential risk of infection for herdsmen, dairy farmers and slaughterhouse workers should not be ignored.

A high CCHFV seroprevalence in cattle in the absence of clinical cases, as we report here, raises several questions as to the underlying eco-epidemiological dynamics of CCHFV in Cameroon and the drivers of disease emergence and clinical infection in the associated human populations. It is likely that clinical cases are being missed due to a milder course of infection or misdiagnosis in the health care setting [10,11,16]. In the studied areas, health care facilities are distant, and people face many difficulties to receive medical care, therefore under-reporting is possible. Anecdotal evidence from discussion with clinicians suggest that fever is a common presenting symptom but, like in many areas in Africa, a lack of diagnostic resources means these cases are treated symptomatically and the causes are never identified. However, it is also possible that human cases are truly absent or occur quite rarely. CCHFV re-emergence after prolonged silent periods has been documented. In Central and East Africa, phylogenetic analysis and serological surveys in human populations have suggested ongoing viral circulation with only a few sporadic cases detected [22,98,99]. While close contact with livestock is one of the strongest risk factors for CCHFV infection in humans, research in endemic and non-endemic locations has introduced contrasting evidence on the relationship between CCHFV exposure rates in livestock and human levels of infection. A multi-causal system involving ecological, environmental, climatic, social, and anthropogenic factors could be responsible of variations on the local viral cycle determining infection trends and human exposure events. A systemic, ‘One health’ view is necessary to unravel the epidemiology of CCHFV at the Cameroonian context.

This study illustrates how serological surveys in cattle can be informative and can contribute to the understanding of CCHFV circulation in settings with scarce epidemiological data. A large representative random sample accounting for the highly hierarchical population structure and a careful analytical approach is one of the main strengths of this study. However, the cross-sectional nature of our data is limited at determining cause-effect relationships, in particular when it comes to environmental and climatic features involved in disease occurrence. CCHFV can induce a strong, long-lasting immune response with an uncertain antibody decline rate. Hence, the possibility of differentiating CCHFV active exposure from passively acquired maternal antibodies or old viral exposures is limited.

Our findings provide baseline data on CCHFV circulation at the North West and Adamawa Regions in Cameroon and its associated factors. The extent to which the local epidemiological picture has changed since data collection remains an open question. In spite of this uncertainty, our results have the potential to create awareness among public health authorities and guide future epidemiological studies under a multi-disciplinary ‘One Health’ approach. An increased risk associated with occupational exposure for pastoralists and dairy keepers is not negligible. Livestock handlers, slaughterhouse workers, veterinarians and other personnel in frequent contact with CCHFV exposed animals should be informed about the risk so that adequate protective measures to avoid transmission are implemented. Upcoming studies should emphasize on quantifying the real impact of the CCHFV circulation for the local population through the combined assessment of viral evidence in ticks, animal hosts and humans. Identifying CCHFV incidence in hospital settings and estimating the prevalence of infection across high-risk populations is a priority. In addition, a deeper understanding of tick-mediated transmission including a better characterization of tick species involved with CCHFV and their distribution is required to clarify local viral dynamics and the risk of infection for human and animal populations. Lastly, molecular characterization of viral strains followed by phylogenetic and phylogeographic analysis are the next step towards a better understanding of viral genetic diversity and dispersion patterns across the country.

## Supporting information

S1 Checklist STROBE Statement—Checklist of items that should be included in reports of cross-sectional studies. (DOC)

S1 Appendix Individual risk-factor model details, performance and fit. (DOCX)

S2 Appendix Ecological model details, performance and fit. (DOCX)

S1 Table Seroprevalence of Crimean-Congo Haemorrhagic fever in pastoral cattle stratified at the Divisional/sub-Divisional level. (DOCX)

S1 Fig Distribution of the seropositive herds in the dairy sample from the North West Region. The map shows the location of sampled herds and its serological status. Each herd is symbolized by a dot and the colour associated to it represents the classification according to the serological status of the herd. Shapefile obtained from GADM database, freely available for academic uses with permission from Global Administrative Areas (https://gadm.org/maps/CMR.html). The figure was made with RStudio version 3.5.3.
(TIFF)
