# Supplementary material for: Seroepidemiology of Crimean-Congo Haemorrhagic Fever among cattle in Cameroon: Implications from a One Health perspective
Source: PLoS Negl Trop Dis. 2022 Mar 21;16(3):e0010217. doi: 10.1371/journal.pntd.0010217 (PMC8936485; doi:10.1371/journal.pntd.0010217)
Supplement: S1 Checklist — (DOC) [file pntd.0010217.s001.doc]

|  | Item No | Recommendation |
| --- | --- | --- |
| **Title and abstract** | 1 | (*a*) Indicate the study’s design with a commonly used term in the title or the abstract – ***Seroepidemiology of Crimean-Congo Haemorrhagic Fever among cattle in Cameroon: Implications from a One Health perspective.***  ***.*** |
|  |  | (*b*) Provide in the abstract an informative and balanced summary of what was done and what was found – ***Abstract and author summary*** |
| Introduction | | |
| Background/rationale | 2 | Explain the scientific background and rationale for the investigation being reported – ***Introduction*** |
| Objectives | 3 | State specific objectives, including any prespecified hypotheses – ***Introduction: Last paragraph*** |
| Methods | | |
| Study design | 4 | Present key elements of study design early in the paper – ***Methods: ‘Study design’ and ‘Serum biobank and associated cattle metadata’*** |
| Setting | 5 | Describe the setting, locations, and relevant dates, including periods of recruitment, exposure, follow-up, and data collection – ***Methods: ‘Background and study setting’, ‘‘Study design’ and ‘Serum biobank and associated cattle metadata’*** |
| Participants | 6 | (*a*) Give the eligibility criteria, and the sources and methods of selection of participants – ***Methods: ‘Study design’ and ‘Serum biobank and associated cattle metadata’*** |
| Variables | 7 | Clearly define all outcomes, exposures, predictors, potential confounders, and effect modifiers. Give diagnostic criteria, if applicable ***– Methods: ‘Data analysis and statistics’*** |
| Data sources/ measurement | 8* | For each variable of interest, give sources of data and details of methods of assessment (measurement). Describe comparability of assessment methods if there is more than one group – ***Methods: ‘Study design’ and ‘Serum biobank and associated cattle metadata’ and ‘Laboratory testing’*** |
| Bias | 9 | Describe any efforts to address potential sources of bias ***– Methods: ‘Data analysis and statistics’*** |
| Study size | 10 | Explain how the study size was arrived at – ***Methods: ‘Study design’*** |
| Quantitative variables | 11 | Explain how quantitative variables were handled in the analyses. If applicable, describe which groupings were chosen and why ***– Methods: ‘Data analysis and statistics’*** |
| Statistical methods | 12 | (*a*) Describe all statistical methods, including those used to control for confounding ***– Methods: ‘Data analysis and statistics’*** |
|  |  | (*b*) Describe any methods used to examine subgroups and interactions ***– Methods: ‘Data analysis and statistics’*** |
|  |  | (*c*) Explain how missing data were addressed – ***NA*** |
|  |  | (*d*) If applicable, describe analytical methods taking account of sampling strategy ***– Methods: ‘Data analysis and statistics’*** |
|  |  | (*e*) Describe any sensitivity analyses – ***NA*** |
| Results | | |
| Participants | 13* | (a) Report numbers of individuals at each stage of study—eg numbers potentially eligible, examined for eligibility, confirmed eligible, included in the study, completing follow-up, and analysed **– *NA*** |
|  |  | (b) Give reasons for non-participation at each stage – ***NA*** |
|  |  | (c) Consider use of a flow diagram – ***NA*** |
| Descriptive data | 14* | (a) Give characteristics of study participants (e.g., demographic, clinical, social) and information on exposures and potential confounders – ***Results: ‘Study population characteristics’*** |
|  |  | (b) Indicate number of participants with missing data for each variable of interest – ***NA*** |
| Outcome data | 15* | Report numbers of outcome events or summary measures – ***Results: ‘Prevalence and spatial distribution of seropositive animals’*** |
| Main results | 16 | (*a*) Give unadjusted estimates and, if applicable, confounder-adjusted estimates and their precision (e.g., 95% confidence interval). Make clear which confounders were adjusted for and why they were included – ***All results*** |
|  |  | (*b*) Report category boundaries when continuous variables were categorized – ***All Results*** |
|  |  | (*c*) If relevant, consider translating estimates of relative risk into absolute risk for a meaningful time period – ***NA*** |
| Other analyses | 17 | Report other analyses done—e.g., analyses of subgroups and interactions, and sensitivity analyses – **NA** |
| Discussion | | |
| Key results | 18 | Summarise key results with reference to study objectives – ***Discussion, first paragraph*** |
| Limitations | 19 | Discuss limitations of the study, taking into account sources of potential bias or imprecision. Discuss both direction and magnitude of any potential bias – ***Discussion, last two paragraphs*** |
| Interpretation | 20 | Give a cautious overall interpretation of results considering objectives, limitations, multiplicity of analyses, results from similar studies, and other relevant evidence – ***All discussion*** |
| Generalisability | 21 | Discuss the generalisability (external validity) of the study results –***All Discussion*** |
| Other information | | |
| Funding | 22 | Give the source of funding and the role of the funders for the present study and, if applicable, for the original study on which the present article is based – ***Acknowledgements and funding*** |

*Give information separately for exposed and unexposed groups.

**Note:** An Explanation and Elaboration article discusses each checklist item and gives methodological background and published examples of transparent reporting. The STROBE checklist is best used in conjunction with this article (freely available on the Web sites of PLoS Medicine at http://www.plosmedicine.org/, Annals of Internal Medicine at http://www.annals.org/, and Epidemiology at http://www.epidem.com/). Information on the STROBE Initiative is available at www.strobe-statement.org.
