# Supplementary material for: Seroepidemiology of Crimean-Congo Haemorrhagic Fever among cattle in Cameroon: Implications from a One Health perspective
Source: PLoS Negl Trop Dis. 2022 Mar 21;16(3):e0010217. doi: 10.1371/journal.pntd.0010217 (PMC8936485; doi:10.1371/journal.pntd.0010217)
Supplement: S1 Appendix — (DOCX) [file pntd.0010217.s002.docx]

**1. Global model**

| CCHF_pn ~ ANISEX + ABREED + AGE + TRACAT + BUYCAT + SHEEPO + DOGGSO + GOATSO + CONBUF + CONHOG + CONOTH + CONANT + strata1 + month + (1 \| strata2/HER_ID)  Where:  CCHF_pn: CCHFV seropositivity (Yes/No)  ANISEX: Sex (Female/Male)  ABREED: Breed (Fulani/Gudali+Crossbreeds)  AGE: Age (Young/Adult/Old)  TRACAT: Going on transhumance (Yes/No)  BUYCAT: Cattle purchase (Yes/No)  SHEEPO: Contact with sheep (Yes/No)  DOGGSO: Contact with dogs (Yes/No)  GOATSO: Contact with goats (Yes/No)  CONBUF: Contact with buffaloes (Yes/No)  CONHOG: Contact with hogs (Yes/No)  CONOTH: Contact with other wildlife animals (Yes/No)  CONANT: Contact with antelopes (Yes/No)  strata1: Study location (North West Region/Vina Division of the Adamawa Region)  strata2: Divisions/Sub-divisions  month: month  HER_ID: Herd |
| --- |

**2. Selected features of candidate models before averaging (delta AIC (Δ_i_) ≤ 2)**

|  | **Candidate models** | **AIC** | **Δ AIC** | **Weight^±^** | **Adjusted ICC ^*^** | **Conditional**  **ICC^**^** | **Tjur’s R^2†^** |
| --- | --- | --- | --- | --- | --- | --- | --- |
| 1 | CCHF ~ ABREED + AGE + BUYCAT + TRACAT | 1568.630 | 0.0000000 | 0.17517754 | 0.286 | 0.198 | 0.3860024 |
| 2 | CCHF ~ ABREED + AGE + BUYCAT + SHEEPO + TRACAT | 1569.460 | 0.8300723 | 0.11567260 | 0.282 | 0.194 | 0.3859728 |
| 3 | CCHF ~ ABREED + AGE + TRACAT | 1569.848 | 1.2176875 | 0.09529299 | 0.295 | 0.206 | 0.3862087 |
| 4 | CCHF ~ ABREED + AGE + SHEEPO + TRACAT | 1569.973 | 1.3429252 | 0.08950884 | 0.289 | 0.200 | 0.3861651 |
| 5 | CCHF ~ ABREED + AGE + BUYCAT + CONBUF + TRACAT | 1570.051 | 1.4212552 | 0.08607098 | 0.286 | 0.197 | 0.3862274 |
| 6 | CCHF ~ ABREED + AGE + ANISEX + BUYCAT + TRACAT | 1570.168 | 1.5380151 | 0.08119003 | 0.287 | 0.199 | 0.3863759 |
| 7 | CCHF ~ ABREED + AGE + BUYCAT + DOGGSO + TRACAT | 1570.218 | 1.5876627 | 0.07919939 | 0.285 | 0.197 | 0.3860188 |
| 8 | CCHF ~ ABREED + AGE + BUYCAT + CONHOG + TRACAT | 1570.329 | 1.6985059 | 0.07492945 | 0.284 | 0.196 | 0.3859191 |
| 9 | CCHF ~ ABREED + AGE + BUYCAT + STRATA + TRACAT | 1570.470 | 1.8402567 | 0.06980263 | 0.286 | 0.198 | 0.3859158 |
| 10 | CCHF ~ ABREED + AGE + BUYCAT + CONANT + TRACAT | 1570.509 | 1.8783887 | 0.06848437 | 0.287 | 0.198 | 0.3860593 |
| 11 | CCHF ~ ABREED + AGE + BUYCAT + GOATSO + TRACAT | 1570.623 | 1.9929686 | 0.06467118 | 0.286 | 0.198 | 0.3859931 |

^±^Akaike weights. Value between 0 and 1 that can be interpreted as the probability that a given model is the best approximating model[1].

^*^ Intraclass correlation coefficient (ICC) for mixed effect models representing the proportion of the variance explained by the hierarchical structured

of the population. Index from 0 to 1. ICC adjusted relates only to the random effects[2].

^**^ ICC conditional considers the fixed effects into the calculation[2].

^†^ Also called Coefficient of discrimination (D), which can be interpreted as the ‘fraction of total variation explained by the model’[3].

**3. Residual diagnostics for hierarchical-mixed logistic regressions using the DHARMA package**[4]**.** Each candidate model before averaging (n = 11) was assessed through: (a) QQ-plot to detect overall deviations from the expected distribution and (b) Plot of residuals against the predicted value. Visual inspection and goodness-of-fit tests (KS, dispersion and outliers) were performed on the simulated residuals; based on these results no deviations from the expected distribution were identified across the candidate models. Equally, no clear pattern in the residuals was recognised, therefore no concerns were raised.

Model 1: CCHF ~ ABREED + AGE + BUYCAT + TRACAT – No problems detected.


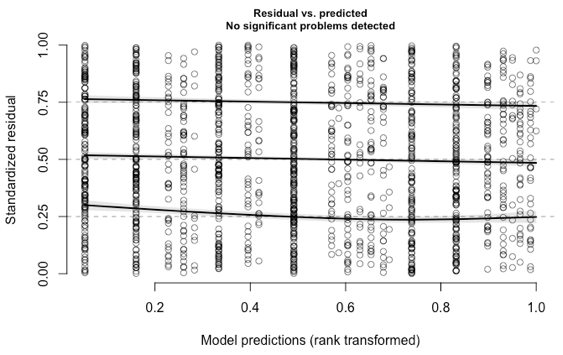

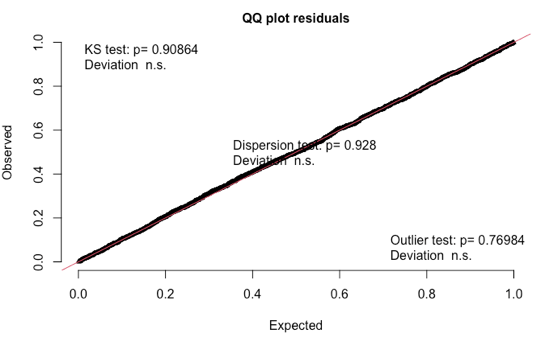


Model 2: CCHF ~ ABREED + AGE + BUYCAT + SHEEPO + TRACAT – No problems detected


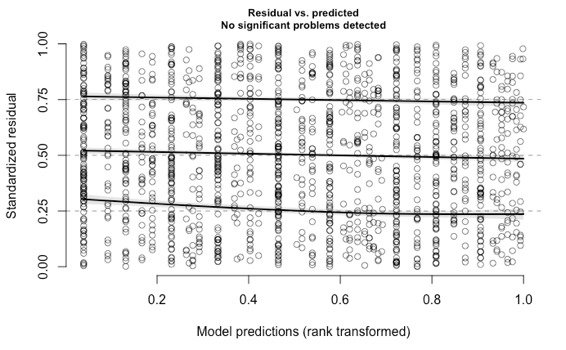

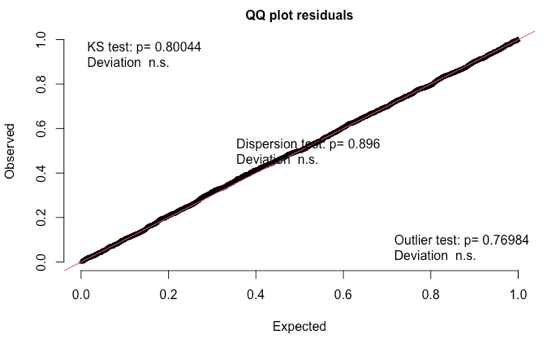


Model 3: CCHF ~ ABREED + AGE + TRACAT – No problems detected


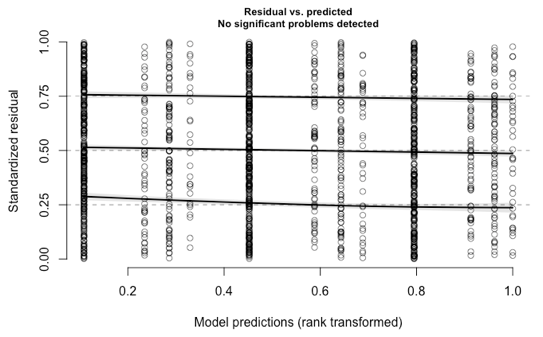

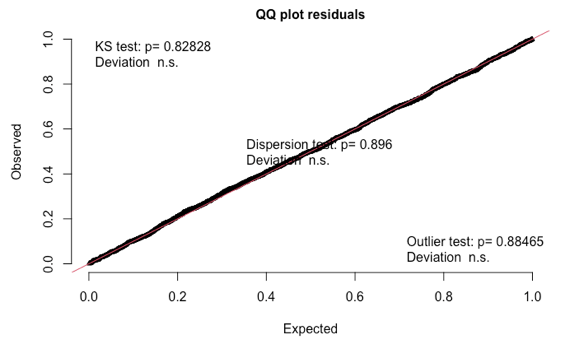


Model 4: CCHF ~ ABREED + AGE + SHEEPO + TRACAT – No problems detected


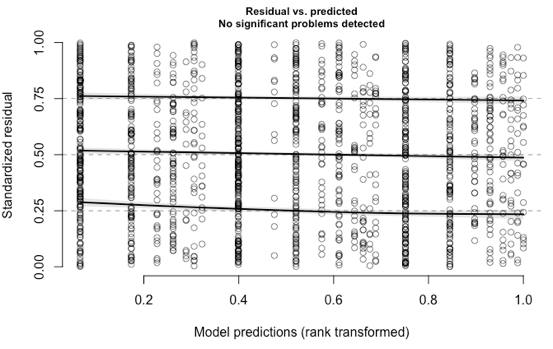

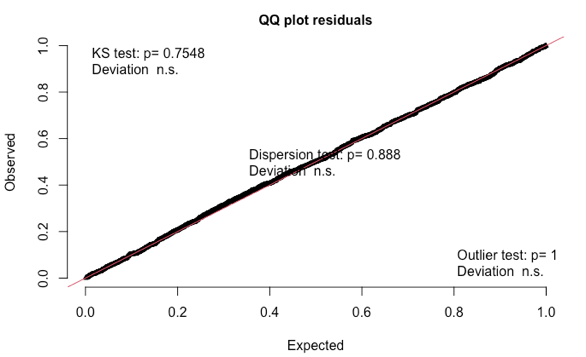


Model 5: CCHF ~ ABREED + AGE + BUYCAT + CONBUF + TRACAT – No problems detected


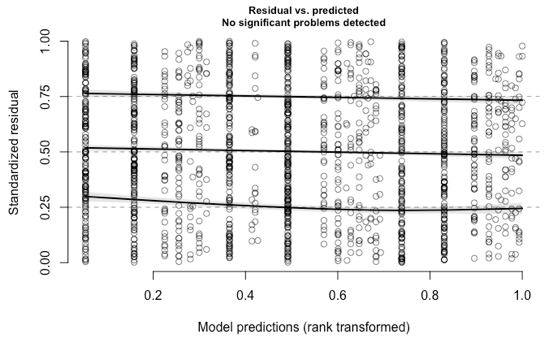

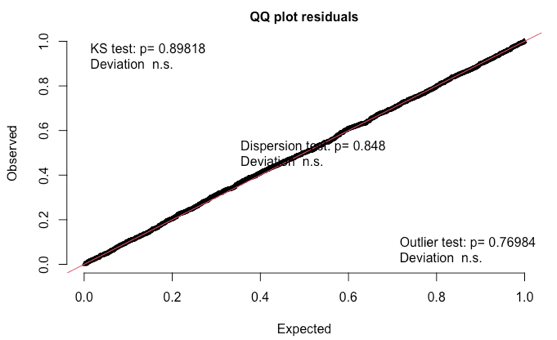


Model 6: CCHF ~ ABREED + AGE + ANISEX + BUYCAT + TRACAT – No problems detected


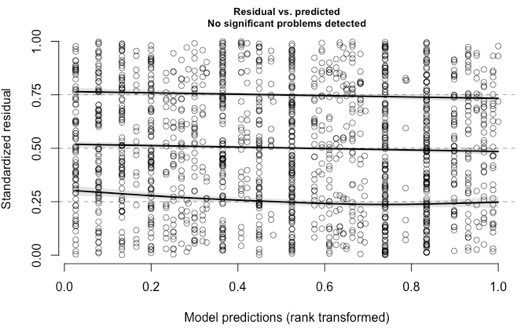

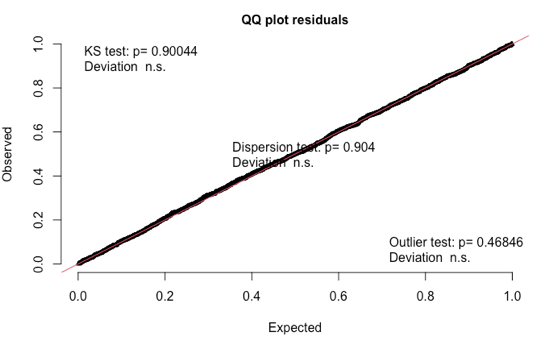


Model 7: CCHF ~ ABREED + AGE + BUYCAT + DOGGSO + TRACAT – No problems detected


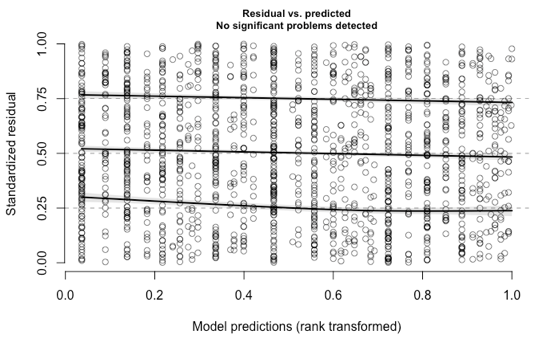

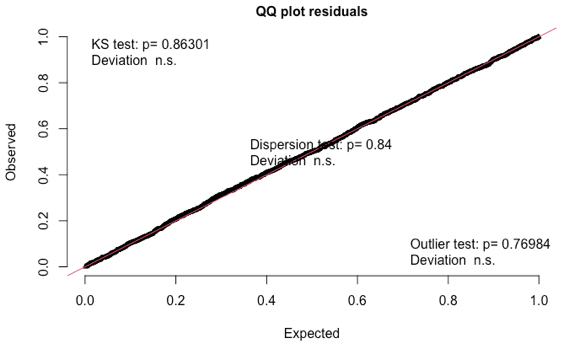


Model 8: CCHF ~ ABREED + AGE + BUYCAT + CONHOG + TRACAT – No problems detected


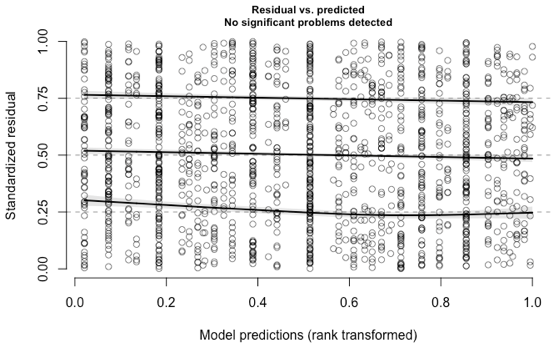

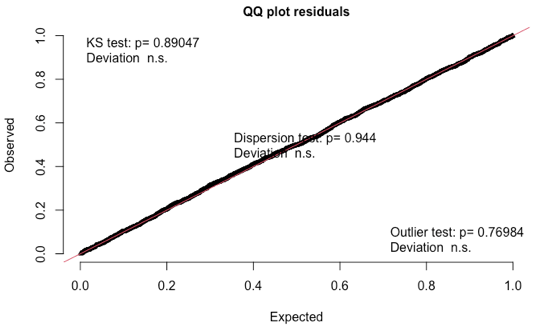


Model 9: CCHF ~ ABREED + AGE + BUYCAT + STRATA + TRACAT – No problems detected


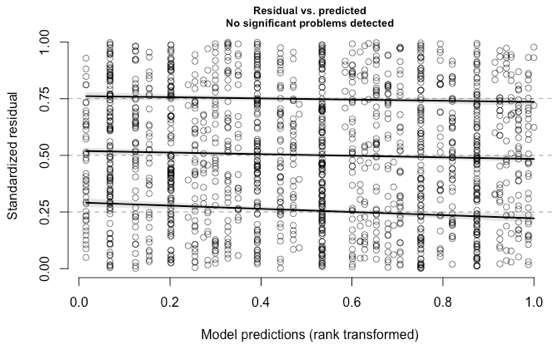

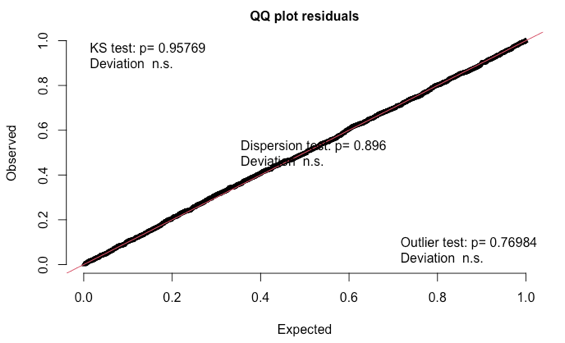


Model 10: CCHF ~ ABREED + AGE + BUYCAT + CONANT + TRACAT – No problems detected


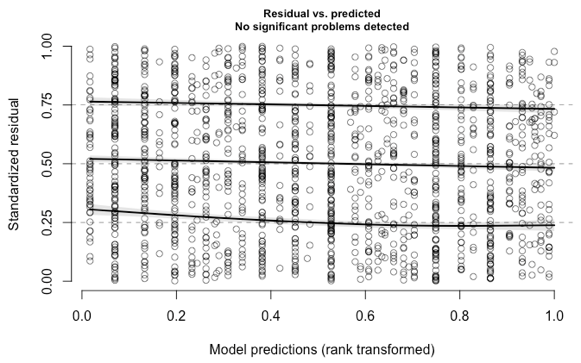

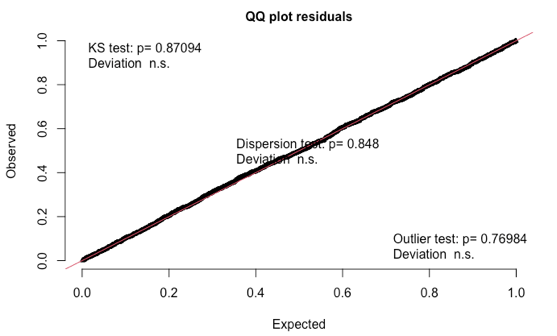


Model 11: CCHF ~ ABREED + AGE + BUYCAT + GOATSO + TRACAT – No problems detected


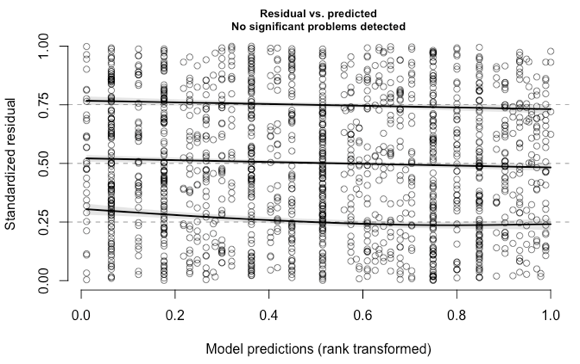

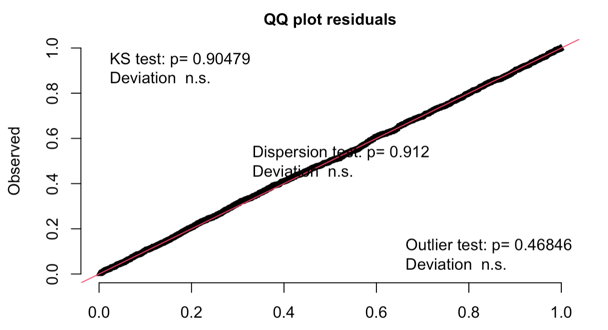


**4. Receiver Operating Characteristic (ROC) curve for the averaged model.** The Area Under the Curve (AUC) summarizes model performance, which in this case is 0.872[5].

**References**

1. Symonds MRE, Moussalli A. A brief guide to model selection, multimodel inference and model averaging in behavioural ecology using Akaike’s information criterion. Behav Ecol Sociobiol. 2011;65: 13–21. doi:10.1007/s00265-010-1037-6

2. Nakagawa S, Johnson PCD, Schielzeth H. The coefficient of determination R2 and intra-class correlation coefficient from generalized linear mixed-effects models revisited and expanded. J R Soc Interface. 2017;14. doi:10.1098/rsif.2017.0213

3. Tjur T. Coefficients of determination in logistic regression models - A new proposal: The coefficient of discrimination. Am Stat. 2009;63: 366–372. doi:10.1198/tast.2009.08210

4. Hartig F. DHARMa: residual diagnostics for hierarchical (multi-level/mixed) regression models. 2021 [cited 23 Jun 2021]. Available: https://cran.r-project.org/web/packages/DHARMa/vignettes/DHARMa.html#calculating-scaled-residuals

5. Sanchez J, Doering M, Multiclass T. Package ‘ pROC .’ 2021. p. 96.
