# Supplementary material for: Seroepidemiology of Crimean-Congo Haemorrhagic Fever among cattle in Cameroon: Implications from a One Health perspective
Source: PLoS Negl Trop Dis. 2022 Mar 21;16(3):e0010217. doi: 10.1371/journal.pntd.0010217 (PMC8936485; doi:10.1371/journal.pntd.0010217)
Supplement: S2 Appendix — (DOCX) [file pntd.0010217.s003.docx]

**1. Univariate models**

|  |  | **OR** | **95% CI** |
| --- | --- | --- | --- |
| Basic Model: CCHF_pn ~ AGE + ABREED + strata1+ (1 \| strata2 / HER_ID) | | | |
| strata1 | North West Region  Vina Division | Reference  1.56 | 0.72 – 3.38 |
| ABREED | Fulani  Gudali/Crossbreed | Reference  0.47 | 0.27 – 0.82 |
| AGE | Young  Adult  Old | Reference  3.16  25.14 | 2.22 – 4.50  16.08 – 39.29 |
| Basic model + ecological variables | | | |
| CCHF_pn ~ AGE + ABREED + strata1 + rh + (1 \| strata2 / HER_ID) |  | 0.001 | 0.00 – 2.08 |
| CCHF_pn ~ AGE + ABREED + strata1 + ah + (1 \| strata2 / HER_ID) |  | 0.60 | 0.00 – 0.87 |
| CCHF_pn ~ AGE + ABREED + strata1 + dtrR + (1 \| strata2 / HER_ID) |  | 684.82 | 0.03 –1.495756e+07 |
| CCHF_pn ~ AGE + ABREED + strata1 + tMeanR + (1 \| strata2 / HER_ID) |  | 0.02 | 0.00 – 1.41 |
| CCHF_pn ~ AGE + ABREED + strata1 + tMinR + (1 \| strata2 / HER_ID) |  | 0.02 | 0.00 – 0.83 |
| CCHF_pn ~ AGE + ABREED + strata1 + tMaxR + (1 \| strata2 / HER_ID) |  | 0.03 | 0.00 – 2.81 |
| CCHF_pn ~ AGE + ABREED + strata1 + tPrecipR + (1 \| strata2 / HER_ID) |  | 0.79 | 0.53 – 1.18 |
| CCHF_pn ~ AGE + ABREED + strata1 + GrassR + (1 \| strata2 / HER_ID) |  | 1.34 | 0.64 – 2.79 |
| CCHF_pn ~ AGE + ABREED + strata1 + TreeR + (1 \| strata2 / HER_ID) |  | 0.63 | 0.23 – 1.68 |
| CCHF_pn ~ AGE + ABREED + strata1 + ShubR + (1 \| strata2 / HER_ID) |  | 2.22 | 1.46 – 3.37 |
| CCHF_pn ~ AGE + ABREED + strata1 + elevRescale + (1 \| strata2 / HER_ID) |  | 1.06 | 0.98 – 1.14 |

CCHF_pn: CCHFV seropositivity (Yes/No); ABREED: Breed (Fulani/Gudali+Crossbreeds); AGE: Age (Young/Adult/Old); tPrecipR: Precipitation (mm/month); tMinR: Minimum temperature (°C); tMeanR: Mean temperature (°C); tMaxR: Maximum temperature (°C); ah: Absolute humidity; rh: Relative humidity; dtrR: diurnal temperature range; elevRescale: Elevation (m); ShrubR: number of pixels (approx 20m*20m) of landcover designated dense shrub within 5km; GrassR: number of pixels (approx 20m*20m) of landcover designated dense grass within 5km; TreeR: number of pixels (approx 20m*20m) of landcover designated dense trees within 5km; strata1: Study location (North West Region/Vina Division of the Adamawa Region); strata2: Divisions/Sub-divisions; HER_ID: Herd

**2. Final model**

| CCHF_pn ~ ABREED + AGE + ah + ShrubR + group + (1 \| strata2/HER_ID)  Adjusted ICC^*^: 0.201  Conditional ICC^**^: 0.131  Tjur’s R^2†^: 0.3711648 |
| --- |

^*^ Intraclass correlation coefficient (ICC) for mixed effect models representing the proportion of the variance explained by the hierarchical structured of the population. Index from 0 to 1. ICC adjusted relates only to the random effects[1].

^**^ ICC conditional considers the fixed effects into the calculation.

^†^ Also called Coefficient of discrimination (D) can be interpreted as the ‘fraction of total variation explained by the model’[2].

**3. Residual diagnostics for hierarchical-mixed logistic regressions using the DHARMA package**[3]**.** The final model was assessed through: (a) QQ-plot to detect overall deviations from the expected distribution and (b) Plot of residuals against the predicted value. Equally, no clear pattern in the residuals was recognised, therefore no concerns were raised.


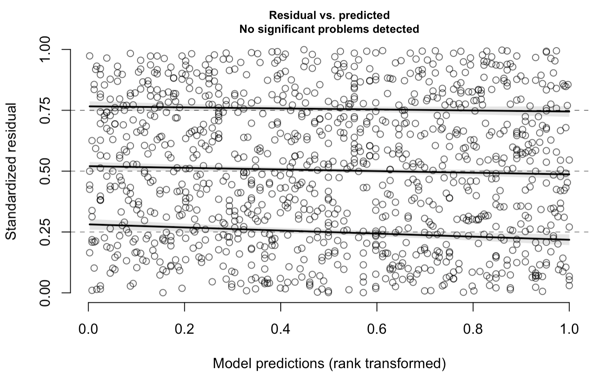

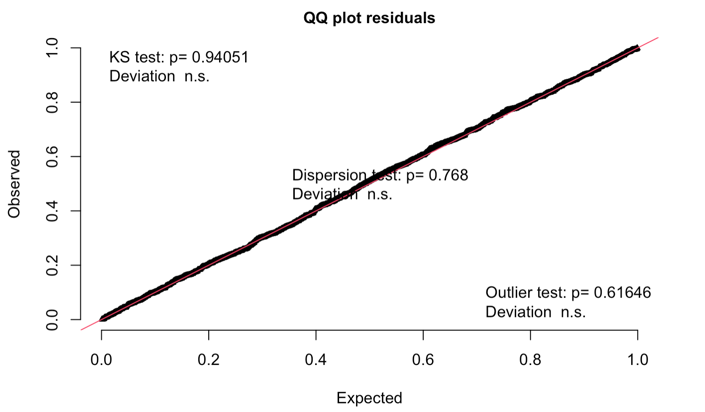


**4. Receiver Operating Characteristic (ROC) curve for the final ecological model.** The Area Under the Curve (AUC) summarizes model performance, which in this case is 0.862[4].


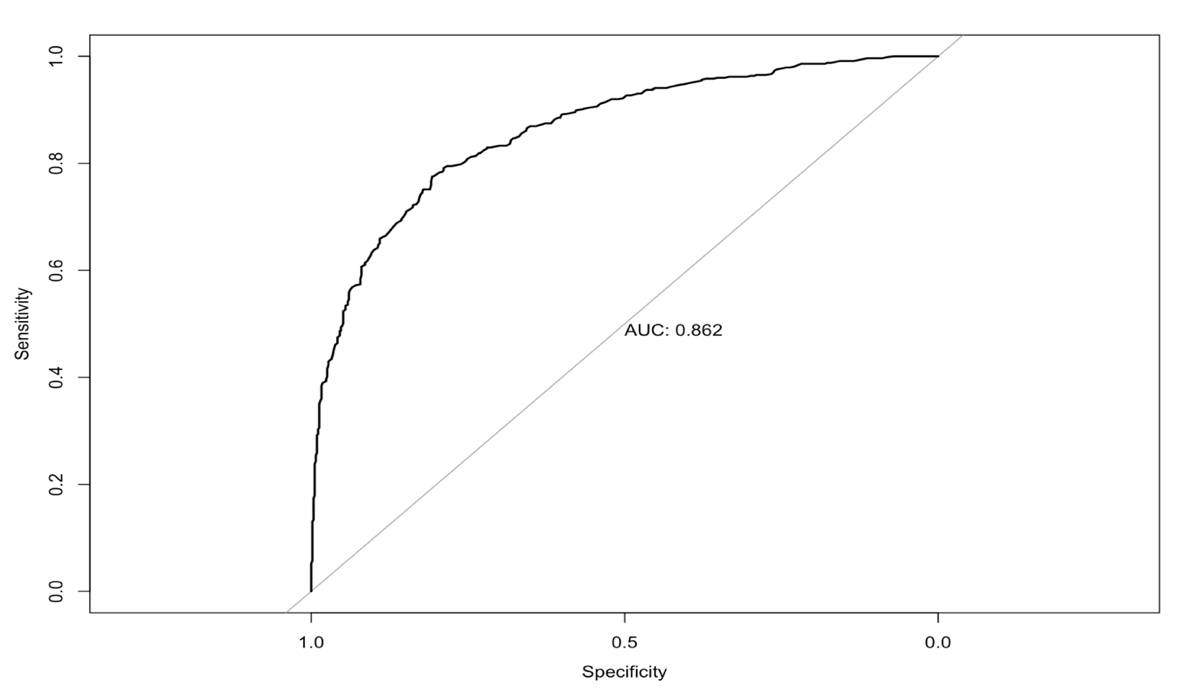


**References**

1. Nakagawa S, Johnson PCD, Schielzeth H. The coefficient of determination R2 and intra-class correlation coefficient from generalized linear mixed-effects models revisited and expanded. J R Soc Interface. 2017;14. doi:10.1098/rsif.2017.0213

2. Tjur T. Coefficients of determination in logistic regression models - A new proposal: The coefficient of discrimination. Am Stat. 2009;63: 366–372. doi:10.1198/tast.2009.08210

3. Hartig F. DHARMa: residual diagnostics for hierarchical (multi-level/mixed) regression models. 2021 [cited 23 Jun 2021]. Available: https://cran.r-project.org/web/packages/DHARMa/vignettes/DHARMa.html#calculating-scaled-residuals

4. Sanchez J, Doering M, Multiclass T. Package ‘ pROC .’ 2021. p. 96.
