# Supplementary material for: Seroepidemiology of Crimean-Congo Haemorrhagic Fever among cattle in Cameroon: Implications from a One Health perspective
Source: PLoS Negl Trop Dis. 2022 Mar 21;16(3):e0010217. doi: 10.1371/journal.pntd.0010217 (PMC8936485; doi:10.1371/journal.pntd.0010217)
Supplement: S1 Table — (DOCX) [file pntd.0010217.s004.docx]

| Location | Division/  Sub-division | Sampled herds | Sampled animals | Adjusted prevalence^*^ |
| --- | --- | --- | --- | --- |
| North West Region | Boyo | 6 | 90 | 44.9 (35.0 – 55.3) |
|  | Bui | 13 | 195 | 66.3 (59.3 – 72.7) |
|  | Donga – Mantung | 12 | 180 | 60.1 (52.7 – 67.0) |
|  | Menchum | 5 | 75 | 62.0 (50.5 – 72.3) |
|  | Mezam | 7 | 105 | 54.8 (45.2 – 64.2) |
|  | Momo | 4 | 60 | 43.8 (31.9 – 56.5) |
|  | Ngo-Ketunjia | 3 | 45 | 71.9 (57.2 – 83.1) |
| Vina Division  (Adamawa Region) | Bélél | 10 | 150 | 64.7 (56.6 – 72.0) |
|  | Martap | 17 | 255 | 38.8 (33.0 – 45.0) |
|  | Mbé | 2 | 30 | 84.2 (67.1- 93.6) |
|  | Nganha | 5 | 73 | 34.6 (24.6 – 46.1) |
|  | Ngaoundéré^**^ | 4 | 60 | 52.2 (39.7 – 64.5) |
|  | Nyambaka | 12 | 180 | 53.9 (46.5 – 61.1) |

^*^ Calculated on the total of animals sampled

^**^ Ngaoundéré I, II, III were collapsed into a single category for sampling and analytical purposes.
