# Supplementary figures and images for: Seroepidemiology of Crimean-Congo Haemorrhagic Fever among cattle in Cameroon: Implications from a One Health perspective
Source: PLoS Negl Trop Dis. 2022 Mar 21;16(3):e0010217. doi: 10.1371/journal.pntd.0010217 (PMC8936485; doi:10.1371/journal.pntd.0010217)

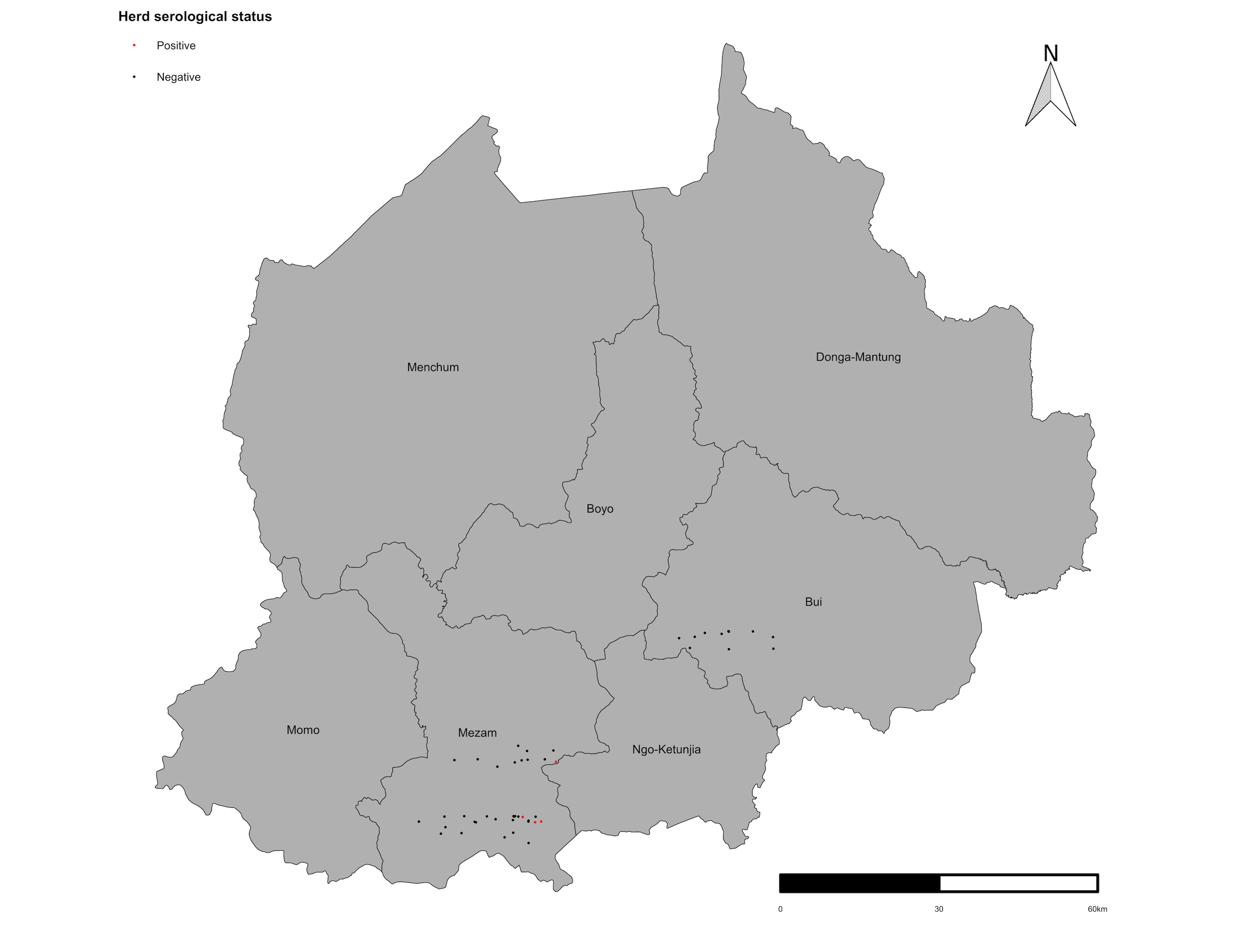

Supplement: S1 Fig — The map shows the location of sampled herds and its serological status. Each herd is symbolized by a dot and the colour associated to it represents the classification according to the serological status of the herd. Shapefile obtained from GADM database, freely available for academic uses with permission from Global Administrative Areas (https://gadm.org/maps/CMR.html). The figure was made with RStudio version 3.5.3. (TIFF) [file pntd.0010217.s005.tiff]
